# Impact of Diabetes and Hyperglycemia on Survival in Advanced Breast Cancer Patients

**DOI:** 10.1155/2012/732027

**Published:** 2012-07-31

**Authors:** Cynthia Villarreal-Garza, Robin Shaw-Dulin, Fernando Lara-Medina, Ludwing Bacon, Daniel Rivera, Lorena Urzua, Christian Aguila, Rebeca Ramirez-Morales, Julieta Santamaria, Enrique Bargallo, Alejandro Mohar, Luis A. Herrera

**Affiliations:** ^1^Clínica de Cáncer de Mama, Instituto Nacional de Cancerología (INCan), 14080 México, DF, Mexico; ^2^Departamento de Oncología Médica, INCan, 14080 México, DF, Mexico; ^3^Unidad de Investigación Biomédica en Cáncer, INCan and Instituto de Investigaciones Biomédicas, Universidad Nacional Autónoma de México, 04510 México, DF, Mexico

## Abstract

*Purpose*. We examined the impact of diabetes and hyperglycemia on cancer-specific survival of patients with metastatic or recurrent breast cancer (BC). *Methods*. We performed a retrospective analysis of 265 patients with advanced BC receiving palliative chemotherapy. BC-specific mortality was compared for diabetic and nondiabetic patients as well as for patients that presented hyperglycemia during treatment. *Results*. No difference was observed between the diabetic and nondiabetic patients in terms of overall survival (OS). A difference in OS was observed between nondiabetic patients and diabetic patients who had hyperglycemia. The OS was greater in diabetic patients with proper metabolic control than diabetic patients with hyperglycemia. The risk of death was higher in patients with mean glucose levels >130 mg/dL during treatment. Several factors were associated with poor OS: tumor stage, hormone-receptor-negative tumors, HER2 negative disease, multiple metastatic sites, presence of visceral metastases, and mean glucose >130 mg/dL. *Conclusion*. Elevated glucose levels are associated with a poor outcome in diabetic and nondiabetic patients in contrast to patients with normoglycemic levels, conferring an elevated risk of death. According to these results, clinicians should monitor glucose levels during treatment for advanced breast cancer disease and take action to maintain normal glucose levels.

## 1. Introduction

Mexico, with a population greater than 100 million, currently has 10 million people with diabetes (types 1 and 2) [1]. Of this group, approximately 2 million are not aware of their condition, and 100,000 people will die from diabetes by the end of this year. Since 2006, breast cancer has been the leading cause of cancer mortality in Mexican women, accounting for 7.6% of female cancer-related deaths [2]. The incidence rate in 2008 was 14.63 per 100,000 women over 15 years of age, and this rate now exceeds that of cervical cancer (10.06 per 100,000 women) [2]. GLOBOCAN predictions for 2030 estimate that 24,386 women will be diagnosed with breast cancer in Mexico and that 9,778 (40.1%) will die from this disease [3]. Diabetes mellitus and breast cancer are major causes of morbidity and death in Mexico and on a global scale.

Recent research has focused attention on the effect of comorbid conditions on all-cause mortality in women with breast cancer [4]. Diabetes, characterized by hyperinsulinemia, insulin resistance, and hyperglycemia, is related to breast cancer. Elevated insulin can directly promote breast cancer cell growth and proliferation, and it can indirectly regulate a variety of factors, including insulin-like growth factors, sex hormones, and adipokines [5].

Data from multiple case-control and cohort studies and two meta-analyses report that women with a history of diabetes have a 15–20% increased risk of breast cancer compared to women without diabetes (RR 1.15–1.20, CI 1.11–1.30) [6, 7]. In patients with breast cancer, diabetes has been associated with adverse outcomes throughout the full course of disease (i.e., initial presentation, treatment, recurrence patterns, and mortality) [7, 8]. In addition, breast cancer patients who are diabetics have a 32% increased risk of chemotherapy-related complications and a 24–61% increased risk of all-cause mortality compared to breast cancer patients without diabetes [9, 10]. 

An analysis of the contribution of diabetes to breast-cancer-specific mortality is difficult because of the substantial mortality attributed to diabetes alone and because diabetes is commonly associated with adverse prognostic factors specific to breast cancer. The purpose of this study was to examine the specific impact of diabetes and hyperglycemia on the cancer-specific survival of patients with metastatic or recurrent breast cancer.

## 2. Patients and Methods

We retrospectively reviewed the clinical records from patients diagnosed with advanced breast cancer who were treated at the National Cancer Institute of Mexico between January 2006 and December 2010. Our analysis included all of the patients with recurrent or newly diagnosed metastatic breast cancers for whom follow-up data and at least three fasting glucose measurements during treatment were available. 

The following clinical and demographic data were obtained for eligible patients from their medical records: age, date of initial diagnosis, clinical stage upon initial diagnosis, pathological characteristics, and date of recurrence or progression. At the time of recurrence, the sites and number of metastatic sites, the type and number of lines of palliative treatment (with the dates of initiation and suspension for each), and any toxicity associated with treatment discontinuation were recorded.

Previous self-reported diagnoses of diabetes or its detection (fasting glucose levels ≥126 mg/dL) at recurrence and whether hypoglycemic treatment was received (and the treatment type) were recorded. The fasting glucose level and the body mass index (BMI) at initiation of each line of palliative therapy were obtained for diabetic and nondiabetic patients. Hyperglycemia was assigned for fasting glucose levels >130 mg/dL.

### 2.1. Statistical Analysis

For descriptive purposes, the continuous variables were summarized as arithmetic means with standard deviations (SDs) and medians with ranges. The categorical variables were summarized as relative frequencies, proportions, and 95% confidence intervals. Pearson Chi-square tests were used to compare the data between diabetic and nondiabetic patients and between patients with and without hyperglycemia. 

Overall survival (OS) was measured from the date of advanced disease to the date of death or last followup. OS was analyzed with the Kaplan-Meier method, and comparisons among subgroups were performed with the log-rank test or the Breslow test. For breast-cancer-specific mortality, a Cox proportional hazards model was used to estimate the hazard ratio (HR) and 95% confidence intervals (95% CI). The a priori variables included in the multivariate analysis were the universally known factors associated with poor outcome (age, tumor stage, nodal status, SBR grade, hormone-receptor status, HER2 status, visceral metastatic involvement, number of metastatic sites, and diagnosis of overweight and obesity at diagnosis of recurrence), the variables of interest in this study (diagnosis of diabetes at recurrent disease, and median glucose >130 mg/dL during palliative treatment), and those variables that showed a difference in the univariate analysis with a *P* < 0.01. The SPSS software (version 17.0; SPSS, Chicago, Ill) was used for data analysis.

## 3. Results

A total of 265 patients receiving palliative therapy were eligible for inclusion. The median age at diagnosis was 49 years (range 22–98 years). The clinical stage at the initial breast cancer diagnosis was distributed as follows: I 10%, IIA 12%, IIB 13%, IIIA 24%, IIIB 16%, IIIC 9%, and IV 16%. Upon inclusion, 84% (225) of the study population had recurrent breast cancer, and 16% (40) had metastatic breast cancer at the initial diagnosis. 

The most common histological findings were invasive ductal carcinoma and lobular carcinoma in 83% and 11% of the cases, respectively. By immunohistochemical analysis, 52.8% of the patients were hormone-receptor-positive, 25.7% had overexpression of HER2, and 24.5% were triple negative. 

In patients with recurrent breast cancer, multiple metastatic sites were identified in 47% of the patients, while 53% had single-site recurrences. The site of initial recurrence was visceral in 62% of the patients and nonvisceral in 38%. The median BMI at recurrence for the study population was 27.4 kg/m^2^ (SD: 4.7). Overweight and obese patients accounted for 42% and 26% of the patients, respectively. A previous diagnosis or detection of diabetes at recurrence was recorded in 40 patients (15%). Pharmacological treatment for diabetes was used by 22 patients (55%), with reported metformin use in 18 of them (45% of diabetic patients).

The differences in the clinical and pathological characteristics at the initial diagnosis or the detection of recurrence between nondiabetic and diabetic patients are shown in Table 1.

Regarding palliative treatment, there was no difference between nondiabetic and diabetic patients. Hormonal palliative treatment was delivered in a comparable proportion between the two groups: 36% versus 43% for the nondiabetic and diabetic patients, respectively (*P* = 0.404). A similar proportion of nondiabetic and diabetic patients received palliative chemotherapy during the course of recurrent disease: 93% versus 98%, respectively (*P* = 0.309). All of the patients with HER2-positive disease received trastuzumab in the palliative setting. Fifty-four percent of the nondiabetic patients received 1 or 2 lines of chemotherapy versus 59% of those with diabetes (*P* = 0.590).

No difference was identified in the proportion of nondiabetic and diabetic patients who experienced toxicity that lead to the suspension of treatment. Grade 3/4 toxicity during palliative treatment was experienced by 14% and 19%, respectively, of the nondiabetic and diabetic patients, which was not significantly different (*P* = 0.253).

All of the deaths were related to breast cancer. The median OS for the entire group was 26.0 months since the diagnosis of recurrence. According to the hormonal and HER2 receptor status, differences were observed as expected for the entire group of patients. For the hormone receptor-positive breast cancer patients, the OS was significantly longer than their counterparts (37.0 versus 18.0 months, respectively, *P* < 0.001). In the triple negative group, the OS was lower compared to the non-triple negative patients (15.0 versus 31.0 months, respectively, *P* = 0.005). As for HER2 status, there was no difference between the patients with HER2-positive and HER2-negative breast cancer (26.0 versus 24.0 months, respectively, *P* = 0.824).

**Figure 1:**
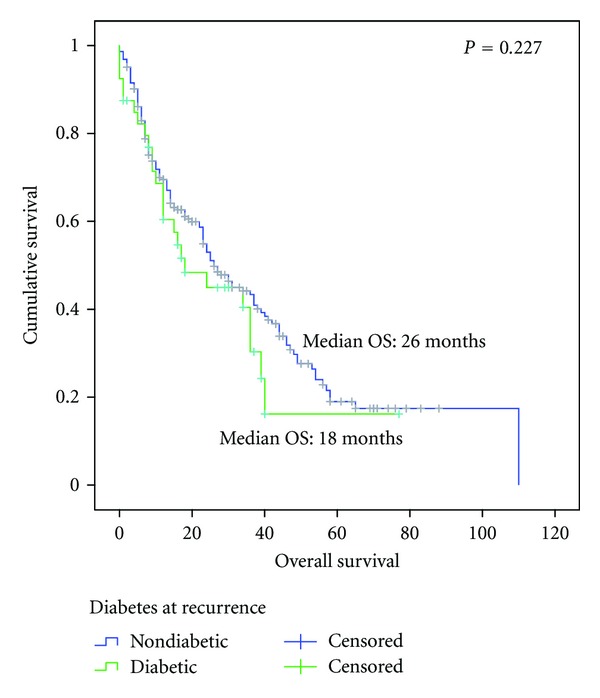
Overall survival since diagnosis of advanced disease between nondiabetic (*n* = 225) and diabetic (*n* = 40) patients.

**Figure 2:**
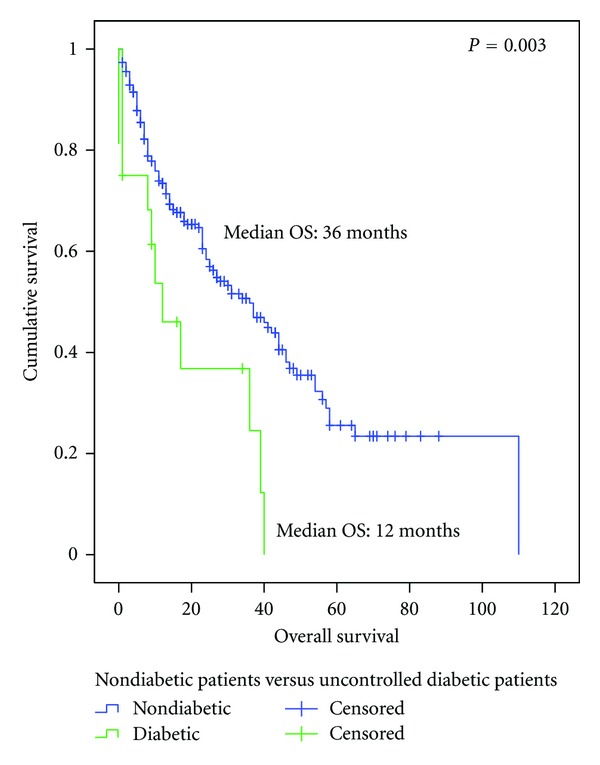
Overall survival since diagnosis of advanced disease between nondiabetic patients (*n* = 225) and uncontrolled diabetic patients (mean glucose >130 mg/dL) (*n* = 16).

For the OS analysis, no difference was observed between the nondiabetic and diabetic patients in terms of OS (26.0 versus 18.0 months, respectively, *P* = 0.227) (Figure 1). However, a statistically significant difference in OS was observed between patients without diabetes and diabetic patients who had hyperglycemia (average fasting glucose level >130 mg/dL), with an OS of 36.0 months versus 12.0 months (*P* = 0.003), respectively (Figure 2).

**Figure 3:**
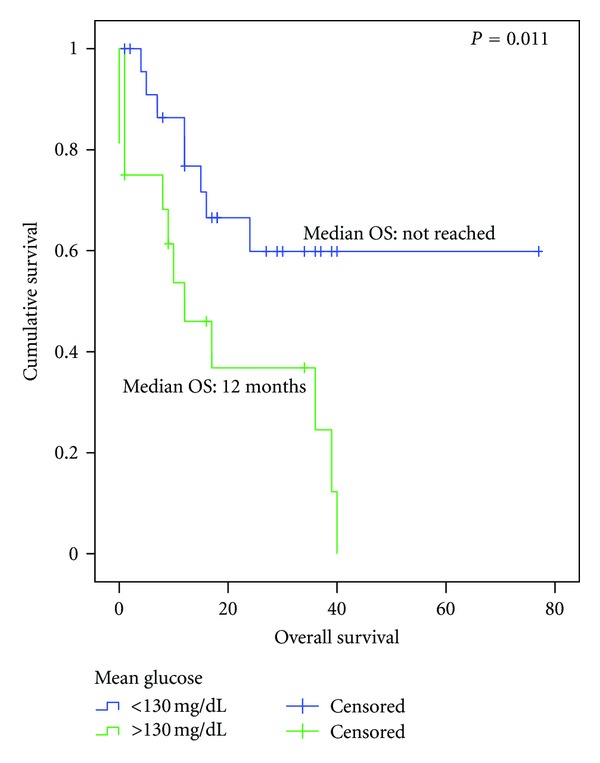
Overall survival since the diagnosis of advanced disease in diabetic patients according to mean glucose >130: normoglycemic (*n* = 24) versus hyperglycemic (*n* = 16).

The OS in diabetic patients with proper metabolic control (average fasting glucose level <130 mg/dL) (*n* = 24) compared to the OS in diabetic patients with hyperglycemia (*n* = 16) was shown to be superior (OS not reached versus 12.0 months, respectively, *P* = 0.01) (Figure 3). The use of metformin showed a nonsignificant benefit in OS in diabetic patients (17.0 months versus 10.2 months metformin-receiving and non-metformin-receiving patients, resp., *P* = 0.371).

**Figure 4:**
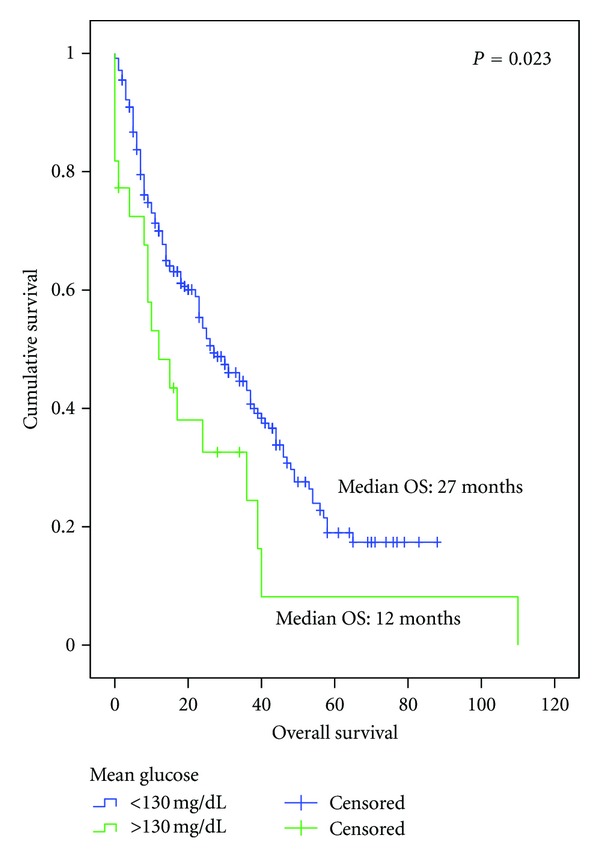
Overall survival since the diagnosis of advanced disease in all of the cohorts according to mean glucose ≤130 (*n* = 243) versus glucose >130 (*n* = 22).

For the entire cohort (diabetic or nondiabetic), patients with mean glucose levels >130 mg/dL during the administration of palliative treatment had a poorer OS compared to patients who did not experience hyperglycemia (OS 27.0 versus 12.0 months, resp., *P* = 0.023) (Figure 4). 

**Figure 5:**
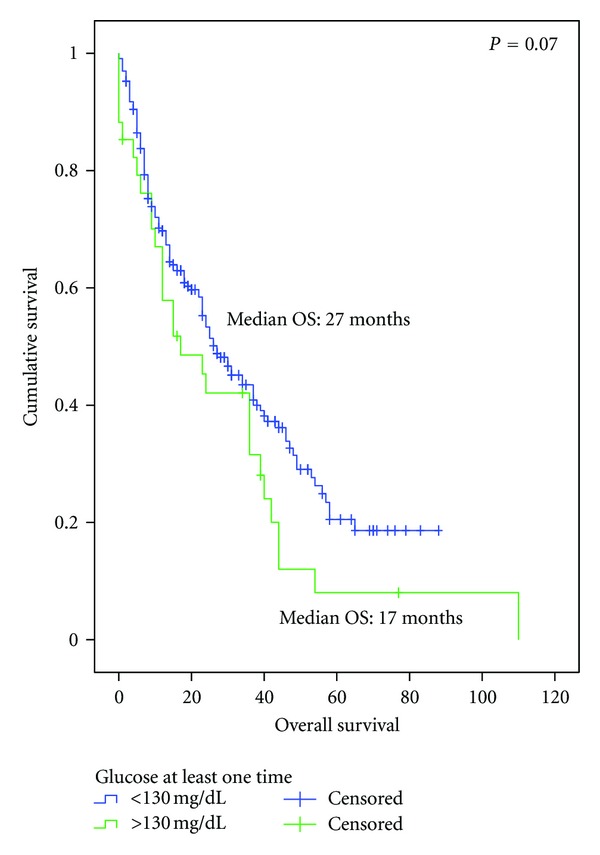
Overall survival since the diagnosis of advanced disease in patients that presented a glucose level >130 mg/dL at least one time during palliative treatment: normoglycemic (*n* = 205) versus glucose >130 (*n* = 60).

Hyperglycemia (fasting glucose level >130 mg/dL) was identified in 32 patients (14.24%) of the nondiabetic population at some point during their treatment. Including the diabetic and nondiabetic subgroups, 60 patients were identified with at least one fasting glucose measurement greater than 130 mg/dL during their palliative treatment. Comparing patients who never experienced hyperglycemia (*n* = 205) to this group, a trend towards a lower OS was observed for patients with hyperglycemia, although this difference did not reached statistical significance (OS 27.0 versus 17.0 months, resp., *P* = 0.07) (Figure 5).

The OS was compared between these two groups according to hormonal and HER2 receptor status. For the hormone-receptor-negative subgroup, there was no difference in the OS between the diabetic and nondiabetic patients. However, for the hormone-receptor-positive subgroup, the nondiabetic subgroup had a significantly longer OS than the diabetic subgroup (41.0 versus 24.0 months, resp., *P* = 0.035). Similar results were found for the HER2 receptor status. No difference was observed in the HER2-negative patients between the diabetic and nondiabetic patients. For the HER2-positive subgroup, a longer OS was observed in the nondiabetic patients than to their counterparts (27.0 versus 9.0 months, resp., *P* = 0.062). When triple-negative status was considered, there was no difference in the OS for the diabetic and nondiabetic patients in either the triple negative or the non-triple negative subgroups. 

The median age at breast cancer diagnosis was 49 for nondiabetic and diabetic patients. The patients were dichotomized according to the median age value: patients <49 and ≥49 years old. No difference was observed among young patients regarding the diagnosis of diabetes. However, for patients older than 49 years, nondiabetic patients had a longer OS compared to diabetic patients (37.0 versus 15.0 months, resp., *P* = 0.043). 

For differences among nondiabetic and diabetic patients according to weight and obesity, patients were dichotomized based on BMIs greater than or lower than 25. No significant difference in OS was observed between the groups.

Table 2 shows data on the OS according to clinical and pathological variables analyzed by univariate and multivariate analyses. In the multivariate analysis, several factors were associated with poor OS, including tumor stage 3/4 (HR 2.7, 95% CI 1.7–4.4, *P* < 0.001), hormone-receptor-negative tumors (HR 0.2, 95% CI 0.1–0.6, *P* = 0.003), HER2 negative disease (HR 0.3, 95% CI 0.1–0.8, *P* = 0.015), multiple metastatic sites (HR 1.6, 95% CI 1.0–2.4, *P* = 0.047), presence of visceral metastases (HR 1.6, 95% CI 1.0–2.5, *P* = 0.042), and mean glucose >130 mg/dL (HR 2.8, 95% CI 1.1–7.3, *P* = 0.034). 

## 4. Discussion

Despite the growing body of evidence indicating that diabetes predicts a poor prognosis after a diagnosis of breast cancer, whether a threshold of glycemic status at which the risk for a poor prognosis significantly increases remains unknown. In our cohort of patients with advanced breast cancer, a diagnosis of diabetes was not associated with a poor outcome. However, when uncontrolled diabetic patients were compared to nondiabetic patients, there was a significant difference in OS, suggesting that poor control of diabetes has a negative impact in patients with metastatic breast cancer receiving palliative treatment. Moreover, diabetic patients with hyperglycemia had a worse prognosis compared to diabetic patients with normal glucose levels.

Hyperglycemia was identified in 14% of nondiabetics at some point while receiving palliative treatment. For patients in either the diabetic or nondiabetic subgroups that experienced hyperglycemia during treatment or who had a mean glucose level greater than 130 mg/dL, a worse outcome was observed compared to normoglycemic patients, with a HR of 1.5 and HR of 2.04 for death, respectively. Erickson et al. recently reported that chronic hyperglycemia (defined as hemoglobin A1C levels ≥6.5%) was independently associated with a statistically significant higher risk of all-cause mortality in early-stage breast cancer survivors, independently of a self-reported diagnosis of diabetes [11]. 

Hyperglycemia may directly influence breast cancer progression and outcomes via several mechanisms, including pathways mediated by high levels of insulin and insulin-like growth factors, sex hormones, and inflammatory markers [11]. Hyperinsulinemia may augment cell proliferation and survival [12, 13]. 

Diabetes has been associated with higher all-cause mortality in women with breast cancer [8]. Several reasons related to the diagnosis of breast cancer in diabetic patients may explain the worse outcomes observed in this group. Women with diabetes may experience a delay in diagnosis, causing them to present with more advanced breast cancer. Because of the concurrent treatment of the chronic diseases associated with diabetes, patients may not undergo routine screening for breast cancer [14]. Furthermore, women with diabetes may receive less aggressive treatment, including chemotherapy, radiotherapy, and/or surgery [8, 15]. The administration of less aggressive treatment may be related to their underlying comorbidities precluding treatment options or a perceived risk of therapy-related toxicity in patients with diabetes [8]. Additionally, women with preexisting diabetes may have a greater risk of chemotherapy-related toxicity, such as infection, fever, and neutropenia. Such risks might explain and justify the use of less aggressive treatments [16, 17]. 

The measurement of substantial mortality attributed solely to diabetes is difficult to assess because diabetes is commonly associated with adverse prognostic factors specific to breast-cancer and other comorbidities. Fleming et al. [18] observed no increase in breast-cancer-specific mortality in patients with diabetes, whereas Srokowski et al. [10] identified increased breast cancer-specific mortality only in patients receiving chemotherapy. The interpretation of these results are further confounded by the findings of Lipscombe et al. [19], who reported a similar mortality in diabetic patients with and without breast cancer. In our cohort, only advanced breast cancer patients were included, thus eliminating possible confounders in prediagnosis delay, clinical prognostic factors or management at initial diagnosis. In addition, all of the patients received palliative treatment for advanced disease, and the deaths were due to breast-cancer-specific causes. Thus, deaths related to other comorbidities did not influence the patients' outcomes. None of the patients abandoned treatment due to treatment-related toxicity, so this risk did not contribute to survival outcome. 

Population studies suggest that metformin decreases the incidence of cancer and cancer-related mortality in diabetic patients [20, 21]. More recently, a retrospective study of patients who received neoadjuvant chemotherapy for breast cancer showed that diabetic cancer patients receiving metformin during their neoadjuvant chemotherapy had a higher pathological complete response rate than diabetic patients not receiving metformin (24% versus 8%, resp., *P* = 0.007) [22]. The antineoplastic effects of metformin in breast cancer are supported by a biological rationale involving important factors associated with breast cancer prognosis. In our study, diabetic patients that received treatment with metformin had a longer OS compared to diabetics with no such treatment. This difference did not reach statistical significance, which was most likely due to the small sample of patients that were managed with this drug. Currently, two-phase I-II trials are ongoing to evaluate the benefit of adding metformin to the treatment of metastatic breast cancer disease. A related phase III trial is being conducted in an adjuvant setting.

Frequently, breast cancer patients treated with chemotherapy receive steroidal agents to avoid or reduce specific adverse events. The hyperglycemia observed in nondiabetic patients while on palliative treatment might be a secondary effect from the use of steroids. Due to the limitations of this study from its retrospective design, we could not associate the rate of hyperglycemia with the use of this type of drug class.

In our study, we observed a worse OS among diabetic patients older than 49 years, a difference that was not observed in younger patients. This finding is consistent with a meta-analysis that showed a stronger association (overall summary RR 1.19, 95% CI 1.15–1.23) among postmenopausal women or among women of postmenopausal age and no significant association for premenopausal women or women of premenopausal age [6]. However, a study in Taiwanese breast cancer patients demonstrated that although a higher risk of breast cancer mortality in diabetic patients occurred in all of the age groups, the magnitude of the risk was largest in the younger age group of 25–54 years [23]. One possible explanation for the discrepancies could be the ethnicities of the study populations because none of the studies were analyzed according to the menopausal status as in the meta-analysis that enrolled Asian women [23].

In conclusion, independent of the cause of hyperglycemia, elevated glucose levels are associated with a poor outcome in diabetic and nondiabetic patients in contrast to patients with normoglycemic levels, conferring an elevated risk of death. According to these results, clinicians should monitor glucose levels during treatment for advanced breast cancer disease and should take action to maintain normal glucose levels.

## Figures and Tables

**Table 1:** Clinical and pathological characteristics in nondiabetic and diabetic patients.

	Nondiabetics	Diabetics	Univariate (*P*)
Median age (Years)	48.0	52.5	0.207
T			
1	27 (12%)	4 (9%)	0.684
2	77 (34%)	12 (29%)	
3	54 (24%)	14 (35%)	
4	67 (30%)	10 (27%)	

N			
0	41 (18%)	9 (23%)	0.695
1	95 (42%)	14 (34%)	
2	56 (25%)	8 (20%)	
3	33 (15%)	9 (23%)	

Grade			
1	29 (13%)	7 (18%)	0.087
2	81 (36%)	20 (49%)	
3	115 (51%)	13 (33%)	

Hormone receptor			
Positive	112 (49%)	28 (70%)	0.018
Negative	113 (51%)	12 (30%)	

HER2			
Positive	60 (27%)	5 (13%)	0.079
Negative	165 (73%)	35 (87%)	

Triple-negative			
Positive	59 (26%)	7 (18%)	0.262
Negative	166 (74%)	33 (82%)	

Metastatic site			
Single	122 (54%)	18 (45%)	0.306
Multiple	103 (46%)	22 (55%)	

Type of metastases			
Nonvisceral	86 (38%)	16 (40%)	0.790
Visceral	139 (62%)	24 (60%)	

Median BMI at recurrence	26.8	27.5	0.359

T: tumor stage according to TNM; N: nodal stage according to TNM; BMI: body mass index.

**Table 2:** Univariate and multivariate analyses according to overall survival.

Variable	Overall survival (median, months)	*P*	HR (95% CI)	*P*
Age (years)			1.2	0.303
Age < 49	26	0.301	(0.8–1.9)	
Age 49	26			

Tumor stage				**<0.001**
1-2	38	**<0.001**	**2.7 (1.7–4.4)**	
3-4	17			

Nodes				0.091
Negative	38	0.276	0.6 (0.4–1.1)	
Positive	24			

Grade				0.083
1-2	39	**0.004**	1.5 (0.9–2.2)	
3	20			

Hormone receptor				**0.003**
Positive	37	**<0.001**	**0.2 (0.1–0.6)**	
Negative	18			

HER2				**0.015**
Positive	26	0.824	**0.3 (0.1–0.8)**	
Negative	24			

Triple-negative				0.072
Positive	15	**0.005**	0.4 (0.1–1.1)	
Negative	31			

Metastatic site				**0.047**
Single	38	**0.018**	**1.6 (1.0–2.4)**	
Multiple	24			

Type of metastases				**0.042**
Nonvisceral	38	**0.019**	**1.6 (1.0–2.5)**	
Visceral	23			

Overweight or obesity at recurrence				0.680
Yes	31	0.310	0.9 (0.6–1.4)	
No	23			

Diabetes at recurrence				0.709
No	26	0.227	0.9 (0.4–1.9)	
Yes	18			

Mean glucose >130				**0.034**
No	27	**0.023**	**2.8 (1.1–7.3)**	
Yes	12			

HR: Hazard ratio.
